# Research on the Preparation of 7N-Grade Ultra-High-Purity Arsenic via Transition-State-Controlled Processes

**DOI:** 10.3390/ma19030545

**Published:** 2026-01-29

**Authors:** Lin Zou, Zhaogang Li, Dachun Liu, Guozheng Zha, Wenlong Jiang

**Affiliations:** 1 National Engineering Research Center of Vacuum Metallurgy, Faculty of Metallurgical and Energy Engineering, Kunming University of Science and Technology, Kunming 650093, China; zoulin0535@163.com (L.Z.); 20210172@kust.edu.cn (G.Z.); wenlong_jiang@kust.edu.cn (W.J.); 2 Shandong Humon Smelting Co., Ltd., Yantai 264000, China; kmustlzg@163.com

**Keywords:** crude arsenic, chlorination reconstruction, distillation purification, hydrogen reduction, 7N high-purity arsenic

## Abstract

To meet the demand for ultra-high-purity arsenic (≥7N) from crude arsenic (As ≥ 99.3%, Sb ≤ 0.6%), an integrated process combining chlorination, distillation and hydrogen reduction was developed. After preliminary purification of crude arsenic by vacuum distillation, chlorine was applied to convert arsenic and its impurities into chlorides. Low-boiling chlorides such as SbCl_3_, S_2_Cl_2_ and Se_2_Cl_2_ were separated by distillation, and ultra-pure AsCl_3_ was finally reduced by hydrogen to obtain ultra-high-purity arsenic. Under optimal conditions—10 mL·min^−1^ Cl_2_ flow, 20 mm–30 mm arsenic particle size and 80 mm–90 mm packing height—the chlorine utilization reached 92.3%. Distillation at 433 K with 4 h total reflux and a 5:1 volumetric reflux ratio yielded AsCl_3_ of 99.99999% purity, with S and Se below 0.02 ppm and 0.01 ppm, respectively. Hydrogen reduction at 1123 K, H_2_/AsCl_3_ molar ratio 1.8 and 623 K condensation temperature achieved an arsenic recovery of 99.13%, a chlorine residue of 20 ppb and a final arsenic purity of 99.9999%. This study provides a feasible route for large-scale production of high-purity arsenic.

## 1. Introduction

Gallium arsenide (GaAs), as the core representative of second-generation semiconductor materials, has become irreplaceable in 5G communications, lasers, infrared detectors and space-grade solar cells because of its high electron mobility (~8500 cm^2^·V^−1^·s^−1^), direct band gap (1.42 eV) and excellent radiation resistance [1,2,3,4]. According to SEMI, the global GaAs wafer market exceeded USD 4.5 billion in 2022, with an annual growth rate of 12.3%. High-purity arsenic (≥7N, 99.99999%) as a critical raw material is required at over 300t per year [5,6,7]. However, domestic production capacity has long been constrained by purification bottlenecks, resulting in an import dependence of 80%, which seriously endangers the security of the semiconductor supply chain.

Currently, industrial high-purity arsenic is mainly produced by chemical vapor deposition (CVD) and zone melting. However, these methods exhibit notable drawbacks: the CVD route requires highly toxic AsH_3_ gas with a wide explosion range (4.5–64%) and shows poor selectivity for Sb, Bi and other congeners (removal < 60%) [8,9,10]; zone melting can upgrade purity to 6N but consumes as much as 300 kWh·kg^−1^ and is sensitive to Sb in the feed (lattice defects increase sharply when Sb > 0.1%) [11,12,13,14,15]. Notably, more than 2000 t per year of crude arsenic (As ≥ 99.3%, Sb ≤ 0.6%) is produced as a non-ferrous smelting by-product in China [16,17,18,19]. Owing to the similar atomic radii of As (1.18 Å) and Sb (1.41 Å) and their close vapor pressures (P_As/P_Sb ≈ 8.3 at 500 °C), conventional vacuum distillation cannot achieve efficient separation (Sb residue > 0.1%), leading to low-value utilization or stockpiling, which not only increases environmental risks but also wastes strategic resources [20,21,22,23,24].

To address these issues, this study proposes a synergistic approach of “chlorination reconstruction distillation purification directed deposition hydrogen reduction”. Selective chlorination is used to convert metal arsenic and impurities into significantly different volatile chlorides (AsCl_3_ b.p. 403.2 K vs. SbCl_5_ b.p. 341 K), indicating that arsenic and antimony have certain differences in the physicochemical properties of their respective chlorides. α-As fillers are introduced to catalyze the conversion of SCl_2_/S_2_Cl_2_ into solid S/Se, eliminating the azeotrope between S/Se chloride and AsCl, thereby achieving effective removal of S/Se. A multi-zone hydrogen reduction furnace with controllable condensation gradient (573 K–623 K) is used for selective deposition of alpha arsenic (crystalline, >90%) and inhibition of amorphous arsenic formation. The thermodynamic mechanism and experimental rules of each step were systematically studied, and the entire process from crude arsenic to 7N ultra-high purity arsenic was established, providing theoretical support and technical paradigm for the preparation of high-purity arsenic.

## 2. Experimental

### 2.1. Experimental Materials

The crude arsenic used in this study was supplied by the comprehensive recovery workshop of a non-ferrous smelter. The crude arsenic used is a composite representative sample obtained from continuous production batches, not a random single batch. The raw materials used in the text are from the same batch of finished materials. Chlorine (industrial grade, ≥99.8%) and hydrogen (ultra-pure, GB/T 3634.2-2011 [25]) were employed.

**Figure 1:**
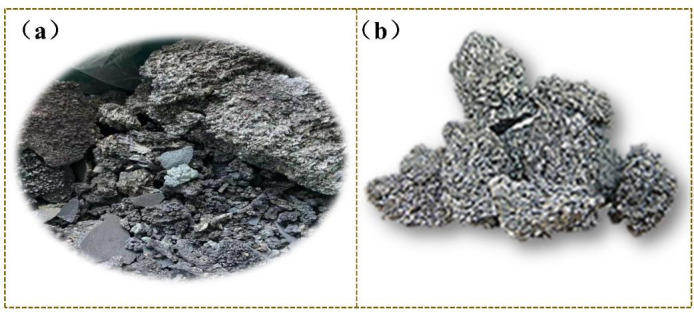
Experimental Materials: (**a**) Macroscopic morphology of crude arsenic samples, (**b**) Localized enlarged view of coarse arsenic sample.

Figure 1 shows the appearance of the crude arsenic sample. The material exhibits a metallic silvery-grey luster and an irregular blocky morphology. Quantitative analysis is given in Table 1. The main component is As (97.28 wt %). Metallic impurities are dominated by Sb (1.688 wt %) and Bi (0.022 wt %), followed by Pb (0.0021 wt %) and Fe (0.0081 wt %). Non-metallic impurities are mainly Se (0.0042 wt %) and S (0.0021 wt %). Owing to the extremely similar physicochemical properties of As, Sb and Bi (all Group VA elements), their deep separation is a common technical challenge in the preparation of high-purity arsenic and thus constitutes the focus of this work.

**Figure 2:**
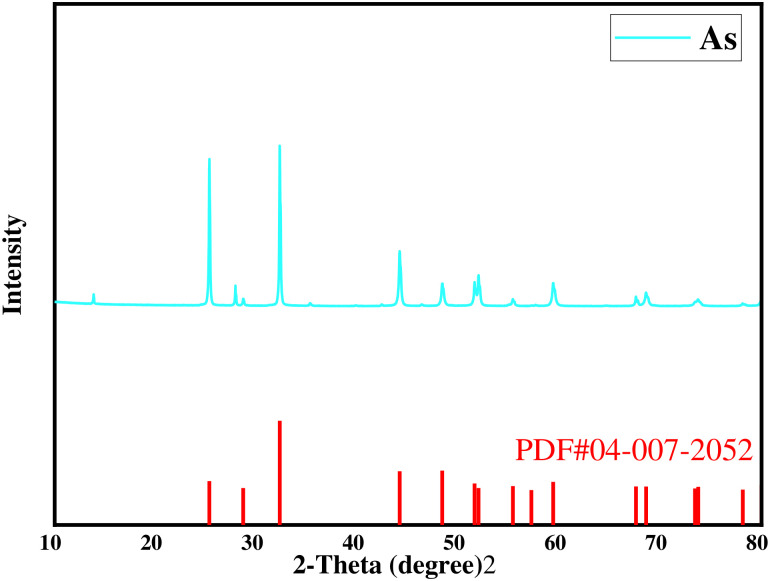
XRD pattern of crude arsenic.

X-ray diffraction (Figure 2) reveals that all discernible peaks correspond to elemental As. The split peak near 2θ = 32° indicates that the crude arsenic is not phase-pure. No distinct arsenic crystalline phases were detected by XRD, which may be attributed to either excessively low concentrations of arsenic-containing impurities (total impurity content < 3 wt %) or their presence in an amorphous/nanocrystalline state.

### 2.2. Experimental Procedure

**Figure 3:**
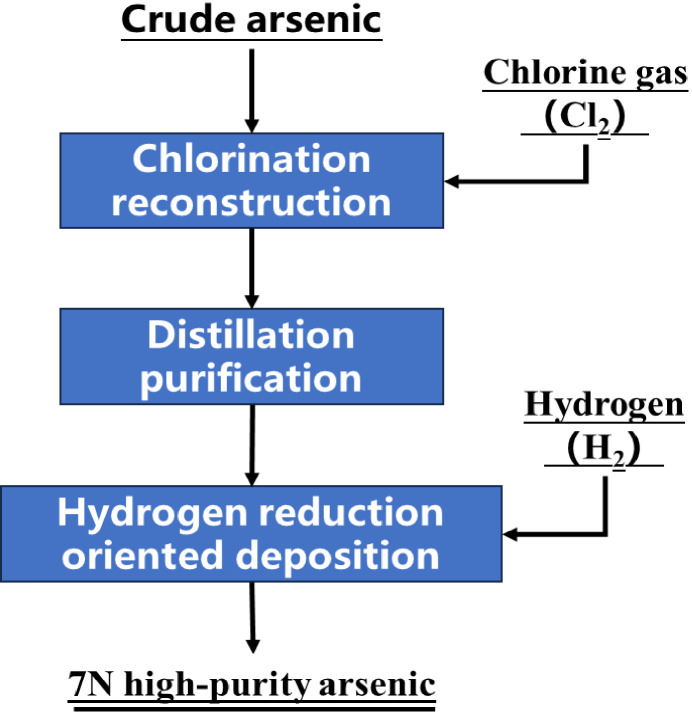
Flowsheet for preparing ultra-high-purity arsenic from crude arsenic.

The proposed process is shown in Figure 3. It comprises chlorination of arsenic to AsCl_3_, distillation purification of AsCl_3_, and hydrogen reduction to high-purity arsenic.

**Figure 4:**
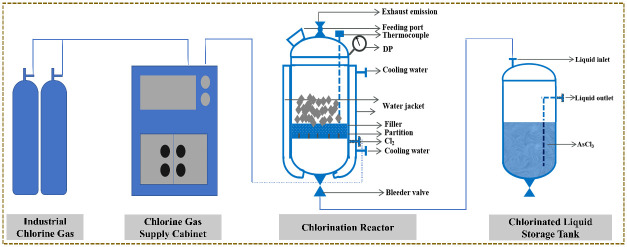
Schematic diagram of chlorination equipment.

Chlorination was carried out in the reactor shown in Figure 4. The reactor is constructed from steel lined with corrosion-resistant material and equipped with an external water jacket for temperature control. Internally, packing and baffles are installed to disperse chlorine and avoid local overheating. The chlorinated liquid storage tank, identical in material, is placed on a balance for mass measurement.

**Figure 5:**
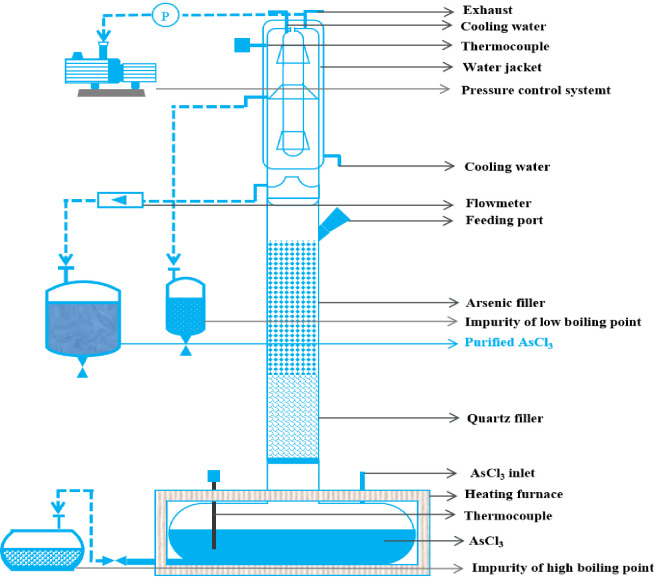
Flowsheet of AsCl_3_ distillation equipment.

Distillation was performed in the quartz packed column shown in Figure 5. The column (20 L–25 L single-batch capacity) consists of a total-condenser head, a quartz-packed section and a horizontal kettle. α-As packing is additionally loaded to catalyze the conversion of S_2_Cl_2_/Se_2_Cl_2_ into solid S/Se.

**Figure 6:**
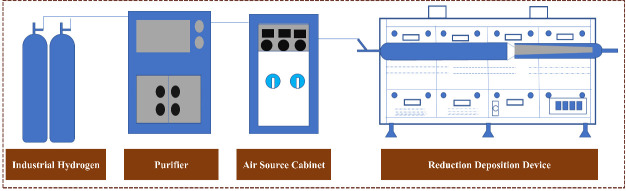
Schematic diagram of AsCl_3_ hydrogen reduction and deposition equipment.

Hydrogen reduction was conducted in the three-zone furnace shown in Figure 6. Temperatures of reduction, transition and deposition zones are independently controlled between 273 K and 1273 K. H_2_/N_2_ flow is regulated and interlocked with furnace parameters.

### 2.3. Characterization

Arsenic grades and impurity contents were determined according to YS/T 43-2011 [26] and YS/T 519.1-2009/519.4-2009 [27]. XRD (Rigaku/D-Max-3c, Tokyo, Japan) and were employed for phase and elemental distribution analyses. The detection instrument was a GD90 Glow Discharge Mass Spectrometer (GD-MS, MSI, London, UK). The samples were prepared using the mixed method of medium-pressure pressing, with high-purity indium as the discharge carrier. High-purity arsenic samples were evenly spread on indium sheets (purity > 99.99999%), pressed into a flat surface using a tablet press, with a compaction thickness of approximately 1 mm, and then placed in sample holders. The instrument was optimized for analysis by adjusting parameters such as argon flow rate (0.75 mL/min), current (1.3 mA), voltage (2.5 V), and integration time (40–100 ms). The detection of the main element arsenic content and impurity elements such as antimony, bismuth, and sulfur was conducted according to the standards YS/T 519.1-2009 and YS/T 519.4-2009. The main element, arsenic, was analyzed using titration, while antimony, bismuth, and sulfur were quantitatively analyzed using ICP-AES (Agilent Technologies 5800, Santa Clara, CA, USA).

## 3. Results and Discussion

### 3.1. Feasibility Theory of Process Route

**Figure 7:**
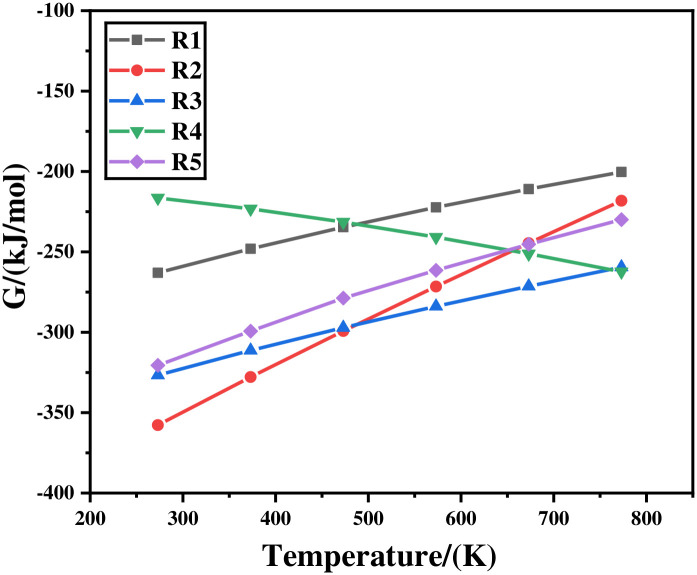
The chlorination reaction result of crude arsenic.

To determine whether the components in crude arsenic reacted during the chlorination experiment, the standard Gibbs free energies (Δ*G*) of different reactions were calculated using Equation (1) [28,29,30,31]:

Δ*G* = Δ*G*_0_ + *RT*In*Q* (1)

where Δ*G*_0_ represents the standard Gibbs free energy in J/mol, and its value corresponds to the conversion of standard reactants to standard products; *R* is the ideal gas constant, whose units are kJ/(mol·K); *T* stands for temperature in Kelvin (K); *Q* is the activity quotient, related to the activities of condensed species and partial pressures of gaseous species. The default reaction activity during the calculation process is 1. The thermodynamics of the chlorination process were investigated based on Gibbs free energy analysis. The reactions of each component during the chlorination process are shown in Table 2, and the relationship between Gibbs free energy and temperature is shown in Figure 7.

**Figure 8:**
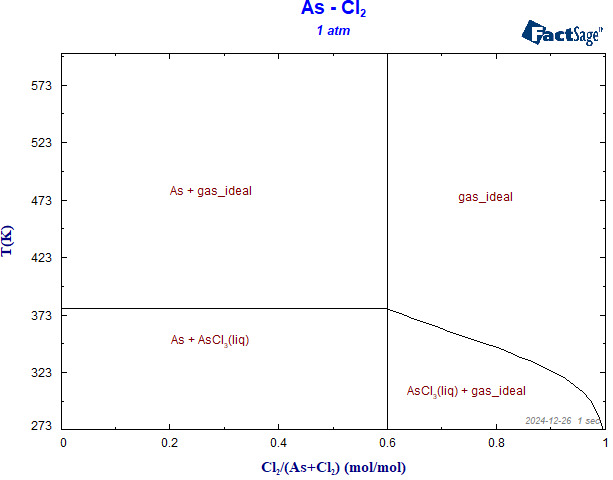
Phase diagram of reaction dominant regions in As-Cl_2_ system.

Standard Gibbs free energies (ΔG) of relevant reactions (Table 2) were calculated. Results (Figure 7) show that ΔG < 0 for R-1 to R-5 in the range 273 K–773 K, indicating spontaneous chlorination of As, Sb and Bi. At 473 K the driving force decreases in the order R-4 > R-1 > R-5 > R-3 > R-2. Notably, SbCl_5_ formed initially is reduced by arsenic to the more stable SbCl_3_. Figure 8 presents the predominance area diagram of the As-Cl_2_ system. When the Cl_2_ fraction is 0.6–1, the only stable condensed phase is AsCl_3_, providing the thermodynamic basis for complete chlorination. The standard Gibbs free energies were obtained from thermodynamic databases integrated in FactSage 7.2, with temperature-dependent heat capacity corrections applied.

As can be seen in Figure 7, the Gibbs free energy of R-1 to R-5 is less than 0 in the reaction temperature range of 273 K–773 K, indicating that Sb and Bi in the crude arsenic on the surface react with Cl_2_. According to the Gibbs free energy calculation, at 473 K, the reaction potential is R-4 > R-1 > R-5 > R-3 > R-2. It is worth noting that SbCl_5_ generated during chlorination reacts with arsenic and transforms into more stable SbCl_3_.

For multiphase systems, the relationship between various aggregation states and their conditions in the multiphase system can be seen from the phase diagram. FactSage 7.2 software was used to construct the predominance area phase diagram of the As-Cl_2_ system components reacting with Cl_2_, as shown in Figure 8. It can be seen in Figure 8 that during the chlorination of arsenic with chlorine, when the proportion of Cl_2_ is less than 0.6, the main stable regions are As element and AsCl_3_. The change in temperature only affects the existence form of As and AsCl_3_. When the proportion of Cl_2_ is between 0.6 and 1, the As stable region disappears, and AsCl_3_ becomes the only stable phase region. The change in temperature mainly affects the excess chlorine in the system and the existence form of AsCl_3_. Therefore, in the process of chlorine chlorination, for safety reasons, the proportion of Cl_2_ should be between 0.6 and 1.

Arsenic reacts with chlorine gas to form chlorinated products, which create a mixed liquid. When this mixed liquid is heated, gas is generated. As the gas leaves the heated area, its temperature gradually decreases. When the gas temperature drops below the boiling point of a certain chlorinated compound in the mixed gas, this chlorinated compound begins to condense, transitioning from a gaseous to a liquid state, thus separating from the mixed gas. Other chlorinated compounds continue to flow since their temperatures have not yet dropped to their boiling points, until the gas temperature reaches the boiling point and begins to condense and separate. This process achieves the separation of different chlorinated compounds through condensation. The characteristic of volatility is defined by the saturated vapor pressure at a specific temperature. Saturated vapor pressure is an indicator of a molecule’s ability to escape from a liquid. The relationship between a substance’s saturated vapor pressure and temperature is illustrated in Equation (2) [32,33,34,35].

logp* = *A/T* + *B*log*T* + *CT* + D (2)

**Figure 9:**
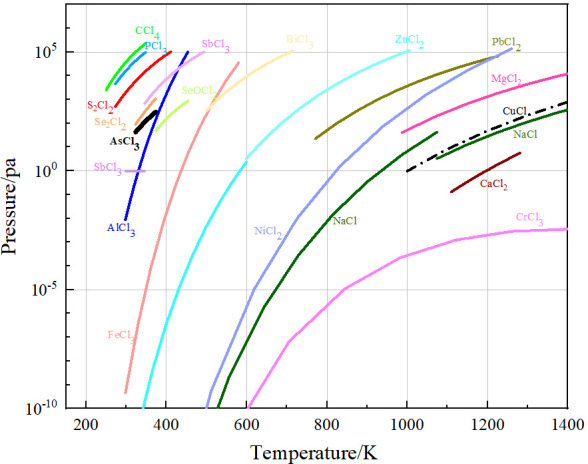
Vapor pressure of each component in arsenic chloride solution.

In the equation, the saturated vapor pressure (Pa) of each component (p*) is calculated using the coefficients *A*, *B*, *C*, and *D*. The temperature (*T*) is measured in Kelvin. Figure 9 illustrates the relationship between temperature and saturated vapor pressure for each component in the chlorination products.

The results shown in Figure 9 indicate that there are significant differences in the melting and boiling points among the components in arsenic chloride solution, as well as notable differences in vapor pressures between the components. The discrepancies in boiling points and vapor pressures among the components provide a theoretical basis for the distillation and condensation separation of arsenic chloride solution. Low-boiling point impurities such as S_2_Cl_2_, Se_2_Cl_2_, CCl_4_, PCl_3_, and SbCl_5_ have relatively high vapor pressures at 400 K, indicating their strong volatility, making them easily vaporized into the gas phase during the distillation process. In contrast, high-boiling-point impurities like AlCl_3_, FeCl_3_, SbCl_3_, BiCl_3_, ZnCl_2_, PbCl_2_, NiCl_2_, MgCl_2_, CuCl_2_, CrCl_3_, and CaCl_2_ have low saturated vapor pressures, and below 473 K, they essentially do not volatilize under standard atmospheric pressure or slight negative pressure. Therefore, controlling the appropriate temperature during the distillation process can effectively achieve the separation of AsCl_3_ from most impurities like SbCl_5_ and AlCl_3_.

**Figure 10:**
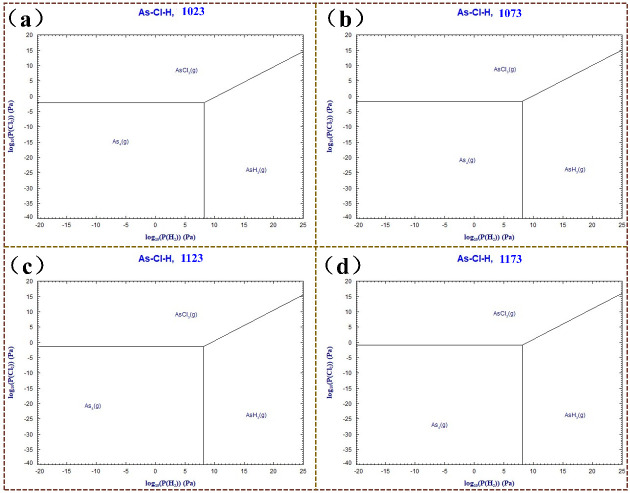
Advantageous regions of As-Cl-H system at different temperatures: (**a**) 1023 K, (**b**) 1073 K, (**c**) 1123 K, (**d**) 1173 K.

The hydrogen potential (lg P(H_2_)/PØ) during the hydrogen reduction process of chlorinated products is a factor affecting the stability of the resulting substances, as shown in Figure 10, along with the hydrogen potential (lg P(H_2_)/PØ) and its variation with temperature. lg P(H_2_)/P_0_ is defined as the decimal logarithm of the ratio of hydrogen partial pressure to standard pressure (1 atm). This dimensionless parameter is used in dominance zone diagrams to represent reducing conditions. The primary system for the hydrogen reduction reaction consists of As and Cl elements, and the hydrogen potential is considered to determine the direction and extent of the reduction reaction. The Predominance Diagrams module of Factsage 7.2 software was used to calculate and construct the predominance area diagram at temperatures ranging from 1023 K to 1223 K, as illustrated in Figure 10.

When a specific thermodynamic parameter, such as the system temperature or a certain partial pressure of a gaseous component, is kept constant, a diagram of the stable regions at isothermal and isobaric conditions can be obtained, depicting the equilibrium of chemical reactions among components in complex systems. The dominance area diagrams of As-Cl-H at different temperatures in Figure 10 indicate that the As-Cl-H system remains stable and comprises only gaseous phases, primarily As(g), AsH_3_(g), and AsCl_3_(g), across various temperatures. As temperature increases from 1023 K to 1223 K, the stability region of AsH_3_(g) diminishes, and that of AsCl_3_(g) also decreases, whereas the As(g) region expands under lower hydrogen potentials. At a temperature of 1123 K, the hydrogen potential in the process of hydrogen reduction that produces the As(g) phase is 6.3. When the hydrogen potential decreases, and the sulfur potential falls below 6.3, the As-Cl-H system tends toward the stability region of As(g). Furthermore, thermodynamic analysis under high-temperature conditions indicates that the preferred reaction forms As(g), followed by the generation of AsH_3_(g) as the hydrogen potential increases. Since AsH_3_(g) is a highly toxic product, its formation should be minimized during the hydrogen reduction process. Therefore, the hydrogen potential of the system should not be excessively high to avoid generating AsH_3_(g).

### 3.2. Experimental Research

**Figure 11:**
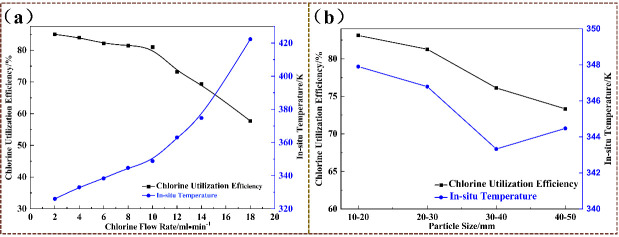
Effects of chlorine flow rate and particle size on experimental results: (**a**) the effect of chlorine gas flow rate on chlorine gas utilization efficiency and temperature, (**b**) the effect of arsenic particle size on chlorine utilization efficiency and temperature.

The reaction between chlorine and arsenic is a self-propagating reaction that releases a significant amount of heat. When arsenic begins to react with chlorine, the reaction immediately releases heat, increasing the system’s temperature. The chlorination reaction process should strictly control the chlorination rate to avoid a violent reaction and excessive system temperature, which could create safety risks. The risk of chlorine arsenic reaction is controlled through multiple measures such as process regulation, spatial isolation, automatic control and monitoring: the PID system controls the flow rate and temperature of chlorine gas at 250–350 °C, dilutes and limits the flow of inert gas; the distance between chlorine gas storage and equipment should be ≥15 m, and the equipment should be placed in a fume hood. The chlorine related components should be made of Hastelloy alloy and equipped with fillers, water jackets, rupture discs, and nitrogen purging; when the interlocking device is abnormal, the chlorine gas will be automatically cut off, and the entire process will be remotely monitored. Personnel will wear positive pressure respirators in the negative pressure isolation room to ensure the safe and effective conduct of the experiment. To investigate the effect of chlorine flow rate on chlorine utilization and system temperature, the chlorination experiment conditions were as follows: chlorine flow rate of 2–20 mL·min^−1^, arsenic particle size of 10 mm–500 mm. The experimental results are shown in kind and in Figure 11.

We calculated the chlorine utilization rate using Equation (3) during the experiment.

(3) $$\eta =\frac{m_{1}\;\times \;58.67\%}{m_{2}}\;\times \;100$$

where: *η* chlorine—utilization rate of chlorine, %;

marsenic trichloride—mass of arsenic trichloride produced, *m*_1_ kg;

mchlorine—mass of chlorine introduced, *m*_2_, kg;

58.67%—mass fraction of chlorine in arsenic trichloride.

As can be seen from Figure 11, increasing the chlorine gas flow rate gradually leads to a decrease in chlorine utilization. When the chlorine gas flow rate is maintained within the range of 2–10 mL·min^−1^, the chlorine utilization rate is 80–85%, and the reactor temperature is 313–353 K. When the flow rate is greater than 10 mL·min^−1^, there is a turning point decrease in chlorine utilization, and the temperature inside the reactor is 420 K. According to the calculated ΔG for reaction R1, an increase in temperature results in a less negative ΔG value, indicating a reduced thermodynamic driving force for AsCl_3_ formation, which is consistent with the decreased chlorination efficiency at higher temperatures. Similarly, examining the effect of arsenic particle size on the reaction system, as the particle size increases, the chlorine utilization rate gradually decreases. When the coarse arsenic particle size is 20–30 mm, the chlorine utilization rate can reach 80.9%, and the reactor temperature is 345 K, which can satisfy the reaction. Therefore, the conditions of chlorine gas flow rate of 4 mL·min^−1^ and coarse arsenic of 20–30 mm are selected as optimized conditions, the reactor temperature is 280 K, and the chlorine utilization rate is 80.9%.

The arsenic chloride solution produced from the chlorination experiment under optimized conditions was detected by ICP-MS, and the composition of impurity elements is shown in Table 3.

The results in Table 3 show that the main impurities in the arsenic trichloride liquid are Sb, Se, and Na. Residual chlorine causes the arsenic trichloride liquid to turn yellow, and the pH value is around 2. It cannot be directly used for the preparation of high-purity arsenic and needs to be purified.

**Figure 12:**
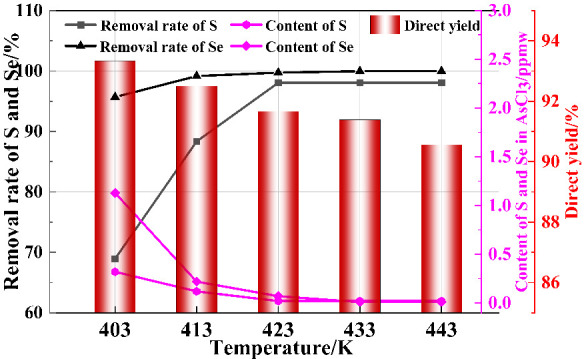
The effect of distillation temperature on the purification of AsCl_3_.

Distillation temperature is a critical parameter in the distillation and purification process, which has a significant impact on the separation of impurities in the AsCl_3_ distillation liquid and the yield of the distillation product. If the distillation temperature is too high or too low, it will cause some impurities to volatilize, affecting the separation and purification effect. Therefore, in order to explore the influence of distillation temperature on the direct recovery rate of AsCl_3_ liquid and the separation effect of impurities, single-factor process experiments were carried out with distillation temperatures controlled at 403 K, 413 K, 423 K, 433 K, and 443 K. Other experimental conditions were as follows: total reflux time 4 h, fractionation rate 30 mL·min^−1^, and volume reflux ratio 5:1. The distillation effect is shown in Figure 12.

As shown in Figure 12, when the rectification temperature increases from 403 K to 433 K, the removal rates of impurities S and Se increase from 68.93% and 95.69% to 98.06% and 99.96%, respectively. After the rectification temperature reaches 443 K, the removal rates of impurities no longer change. At this point, the content of impurities S and Se in the purified AsCl_3_ liquid drops to below 0.02 ppm and 0.01 ppm, respectively, both of which are lower than the detection limit of ICP-MS. The direct recovery rate of the purified AsCl_3_ liquid remains around 91.67%, indicating that increasing the rectification temperature is beneficial for the separation of impurity elements S and Se from AsCl_3_. At 433 K, the impurity elements S and Se in the AsCl_3_ liquid have been completely removed. Continuing to increase the temperature will lead to a decrease in the direct recovery rate of the product and waste of excess energy. Therefore, a rectification temperature of 433 K is chosen as the optimal temperature.

**Figure 13:**
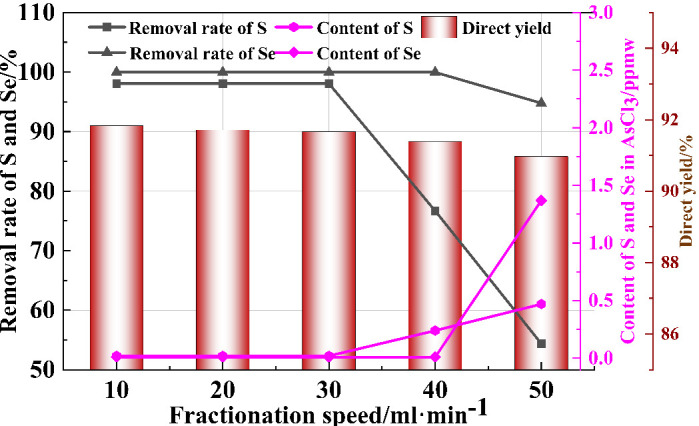
The effect of fractionation speed on the purification of AsCl_3_.

The fractionation rate directly affects the condensation and separation effect of arsenic chloride liquid and impurities, and also has a great impact on the volatilization efficiency of arsenic chloride liquid. Therefore, in order to study the influence of the fractionation rate on the direct recovery rate of AsCl_3_ liquid and the separation effect of impurities, single-factor process experiments were carried out with fractionation rates of 10 mL·min^−1^, 20 mL·min^−1^, 30 mL·min^−1^, 40 mL·min^−1^, and 50 mL·min^−1^. Other experimental conditions were: total reflux time of 4 h, rectification temperature of 433 K, and volume reflux ratio of 5:1. The rectification effect is shown in Figure 13.

As shown in Figure 13, when the fractionation speed increases from 10 mL·min^−1^ to 50 mL·min^−1^, the removal rates of impurity elements S and Se decrease from 98.06% and 99.96% to 40.78% and 96.45%, respectively. This indicates that an excessively high fractionation speed inhibits the removal of S and Se, mainly because the excessively high separation speed prevents complete condensation and separation of the fractions, leading to incomplete fractionation of S and Se from the main component AsCl_3_, resulting in a poor removal effect of S and Se. When the fractionation speed is 30 mL·min^−1^, the removal effect of impurity elements S and Se and the direct recovery rate of AsCl3 liquid are both relatively ideal. Therefore, a fractionation speed of 30 mL·min^−1^ is selected as the optimal fractionation speed. At this time, the S content in the refined AsCl_3_ liquid is less than 0.02 ppm, and the Se content is less than 0.01 ppm.

**Figure 14:**
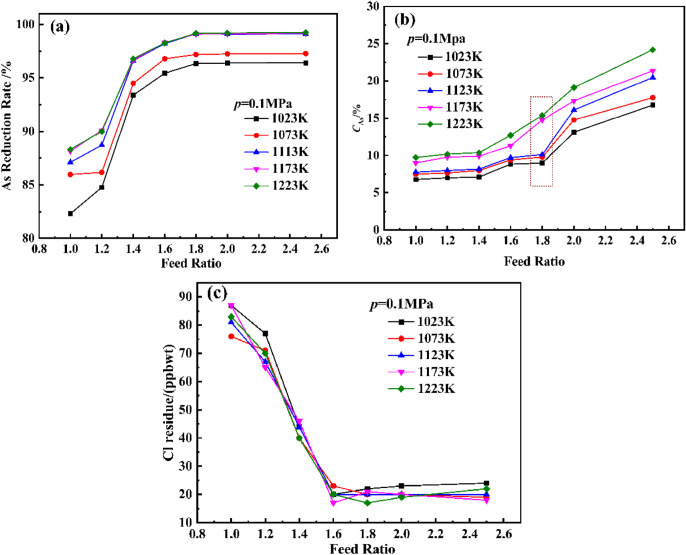
Illustrates the effects of feed ratio and temperature on (**a**) arsenic reduction rate; (**b**) amorphous arsenic proportion; and (**c**) Cl residual content.

To investigate the effect of different feed ratios and reduction temperatures on the hydrogen reduction of arsenic chloride, the feed ratios were set to 1.0, 1.2, 1.4, 1.6, 1.8, 2.0, and 2.5, and the reduction temperatures were set to 1023 K,1073 K, 1123 K, 1173 K, and 1223 K. Other experimental conditions were a condensation temperature of 350 °C and a hydrogen pressure of 0.1 MPa. The results are shown in Figure 14.

According to Figure 14a, as the reduction temperature increases from 1023 K to 1123 K, the reduction rate of arsenic gradually increases. However, when the reduction temperature continues to increase to 1223 K, there is no significant increase in the reduction rate of arsenic. At the same time, the arsenic reduction rate increases with the increase in feed ratio. When the feed ratio reaches 1.8, continuing to increase the feed ratio does not increase the arsenic reduction rate. From Figure 14b, it can be seen that the proportion of amorphous arsenic gradually increases with the increase in temperature. After a reduction temperature of 1123 K and a feed ratio of 1.8, the proportion of amorphous arsenic rapidly increases. Figure 14c shows that the feed ratio has a greater impact on the residual Cl content compared to the reduction temperature. At a reduction temperature of 1123 K, as the feed ratio increases, the residual Cl content gradually decreases from 81 ppb to 20 ppb. From the above analysis, it can be concluded that the experimental results are consistent with the theoretical analysis in Section 3.1. The reduction temperature and excess feed ratio (excess hydrogen) are conducive to the reduction of arsenic and the removal of residual Cl elements in the reduced arsenic. However, they may lead to the production of excess amorphous arsenic. Therefore, a reduction temperature of 1123 K and a feed ratio of 1.8 were selected. At this time, the arsenic reduction rate is 99.13%, the proportion of amorphous arsenic is 10.11%, and the residual amount of Cl is 20 ppb. According to the above experimental results, high-purity arsenic was produced under the optimal process conditions of reduction temperature of 1123 K, feed ratio of 1.8, pressure of 0.1–0.15 MPa, and condensation temperature of 623 K. The GDMS assay results of high-purity arsenic are compared with industry standards in Table 4. GDMS testing shows that the purity of high-purity arsenic in the product meets the standard of 7N high-purity arsenic in YS/T 43-2011.

## 4. Conclusions and Future Perspectives

This article provides an in-depth analysis of the chlorination reconstruction distillation purification hydrogen reduction directional deposition process for crude arsenic, and draws the following conclusions.

(i) Theoretical studies have shown that the Gibbs free energy of the reaction is less than 0, indicating that arsenic and major impurities can theoretically react directly with Cl_2_ to generate corresponding chlorides. AlCl_3_, SbCl_3_, and BiCl_3_ high boiling point impurities have low saturated vapor pressures. When the temperature is below 473 K, controlling the temperature can effectively achieve the separation of AlCl_3_ from most impurities. The phase-dominant region diagram of the As-Cl-H system indicates that increasing the temperature is beneficial for the reduction of AsCl_3_ to As by hydrogen gas, and the reduction process by hydrogen gas is less affected by temperature.

(ii) Experimental studies have shown that controlling the experimental conditions during the chlorination reconstruction stage with arsenic particles of 20 mm–30 mm, fillers of 80 mm–90 mm, and a Cl_2_ flow rate of 10 mL·min^−1^ can achieve a Cl_2_ utilization rate of 83.6%; under the conditions of controlled distillation at 433 K, 30 mL·min^−1^ fractionation, full reflux for 4 h, and reflux ratio of 5:1, the S removal rate can reach 98.1%, the Se removal rate can reach 99.96%, and the AsCl_3_ purity can reach 99.99999%; under the conditions of a hydrogen reduction temperature of 1123 K, a feed ratio of H2/AsCl = 1.8, 0.1 MPa, and a condensation temperature of 623 K during the directional deposition stage of hydrogen reduction, an arsenic reduction rate of 99.1% can be achieved, and the arsenic purity meets the standard of 7N high-purity arsenic in YS/T 43-2011.

This process has connected the complete technical chain from 4N crude arsenic to 7N ultra-high purity arsenic, providing a solution for the security of high-purity semiconductor material supply chain.

## Figures and Tables

**Table 1:** Quantitative chemical composition of crude arsenic (wt %).

**Element**	**As**	**Sb**	**Bi**	**Fe**	**K**	**Ca**	**Pb**
Content	97.28	1.688	0.022	0.0081	0.0064	0.004	0.0021
**Element**	**Na**	**Al**	**Zn**	**Mg**	**Cu**	**Se**	**S**
Content	0.0009	0.0006	0.0003	0.0001	0.0001	0.0042	0.0021

**Table 2:** The chemical equation for the chlorination reaction of elemental arsenic at 473 K.

Number	Reaction	Δ*G*/(kJ/mol)	∆r*G*m⊖/(kJ/mol)
R-1	2As + 3Cl_2_(g) = 2AsCl_3_(l)	−234.601	−263.013
R-2	2Sb(s) + 5Cl_2_(g) = 2SbCl_5_(l)	−299.227	−357.701
R-3	2Sb + 3Cl_2_(g) = 2SbCl_3_(l)	−297.112	−326.648
R-4	SbCl_5_(l) + As(s) = SbCl_3_(l) + AsCl_3_(l)	−231.43	−216.433
R-5	Bi(s) + Cl_2_(g) = BiCl_3_(s)	−278.747	−320.541

**Table 3:** Composition of impurity elements in AsCl_3_ solution (ppm).

**Element**	**Na**	**Mg**	**Al**	**K**	**S**	**Ca**	**Cr**
Content	1.65	0.24	0.07	2.03	1.03	1.09	0.03
**Element**	**Fe**	**Ni**	**Zn**	**Pb**	**Cu**	**Se**	**Ag**
Content	0.054	0.017	2.22	0.14	0.054	26.23	0.001
**Element**	**Sb**	**Bi**					
Content	1279.86	2.17					

**Table 4:** Detection results of high purity arsenic by GDMS.

Element	Element Content (ppm)	YS/T 43-2011 Standard (ppm)
As	>99.99999	>99.99999
Na	<5	<10
Mg	<5	<5
Al	<5	<5
K	<5	<10
Ca	<5	<10
P	<5	<5
Cr	<5	<10
Fe	<5	<10
Zn	<5	<5
Ag	<3	<10
Sb	<5	<10
Pb	<5	<5
Bi	<5	<10
Ni	<5	<10
Cu	<2	<5
Ti	<5	<10

## Data Availability

The original contributions presented in this study are included in the article. Further inquiries can be directed to the corresponding author.
